# Ablation of Tak1 in osteoclast progenitor leads to defects in skeletal growth and bone remodeling in mice

**DOI:** 10.1038/srep07158

**Published:** 2014-11-24

**Authors:** Bing Qi, Qian Cong, Ping Li, Gang Ma, Xizhi Guo, James Yeh, Min Xie, Michael D. Schneider, Huijuan Liu, Baojie Li

**Affiliations:** 1 School of Biological Science, Taishan Medical University, Shandong, China; 2 Bio-X Institutes, Key Laboratory for the Genetics of Developmental and Neuropsychiatric Disorders, Ministry of Education, Shanghai Jiao Tong University, Shanghai, China; 3 Department of Medicine, UT Southwestern Medical Center, Dallas, Texas, USA; 4 National Heart and Lung Institute, Imperial College London, London, United Kingdom

## Abstract

Tak1 is a MAPKKK that can be activated by growth factors and cytokines such as RANKL and BMPs and its downstream pathways include NF-κB and JNK/p38 MAPKs. Tak1 is essential for mouse embryonic development and plays critical roles in tissue homeostasis. Previous studies have shown that Tak1 is a positive regulator of osteoclast maturation, yet its roles in bone growth and remodeling have not been assessed, as mature osteoclast-specific Tak1 deletion with Cstk-Cre resulted in runtedness and postnatal lethality. Here we generated osteoclast progenitor (monocyte)-specific Tak1 knockout mice and found that these mice show normal body weight, limb size and fertility, and osteopetrosis with severity similar to that of RANK or RANKL deficient mice. Mechanistically, Tak1 deficiency altered the signaling of NF-κB, p38MAPK, and Smad1/5/8 and the expression of PU.1, MITF, c-Fos, and NFATc1, suggesting that Tak1 regulates osteoclast differentiation at multiple stages via multiple signaling pathways. Moreover, the Tak1 mutant mice showed defects in skull, articular cartilage, and mesenchymal stromal cells. Ex vivo Tak1−/− monocytes also showed enhanced ability in promoting osteogenic differentiation of mesenchymal stromal cells. These findings indicate that Tak1 functions in osteoclastogenesis in a cell-autonomous manner and in osteoblastogenesis and chondrogenesis in non-cell-autonomous manners.

Bone related diseases, in particular osteoporosis and osteoarthritis, affect hundreds of million people worldwide^1^,^2^,^3^. Osteoporosis is caused by disruption of the balance between bone formation, which is carried out by osteoblasts, and bone resorption, which is carried out by osteoclasts^4^. Under normal conditions, bone resorption and formation are coupled at multiple levels, and coordination between these two events is essential for maintaining proper bone mass and for preventing the development of metabolic bone diseases such as osteopetrosis and osteoporosis. For example, mesenchymal stem cells (MSC) and osteoblasts synthesize M-CSF, RANKL, and OPG to regulate osteoclastogenesis^5^,^6^. Yet how bone resorption communicates with formation is not completely understood^7^.

Osteoclasts are large multinucleated cells that secrete matrix proteinases including Cathepsin K (encoded by *Cstk*), Tartrate-resistant acid phosphatase (TRAP) into resorption pits to degrade collagens and other matrix proteins^3^,^8^. Osteoclasts are derived from the hematopoietic monocyte lineage, which can give rise to macrophage and neutrophils as well. Osteoclast differentiation from monocyte is a multiple-step process that requires distinct signaling molecules and transcription factors at various stages^9^,^10^. Transcription factor PU.1 plays a role in early monocyte differentiation and stimulates the expression of macrophage colony-stimulating factor (M-CSF). Deletion of PU.1 in mice results in a complete lack of macrophages and osteoclasts and causes osteopetrosis^11^. M-CSF can bind to its receptor and stimulate proliferation of monocytes^12^,^13^. RANKL, which can be provided by MSCs and osteoblasts, promotes monocyte differentiation into osteoclasts^14^. RANKL binds to RANK molecules located on monocyte surface, leading to Tak1 (TGFβ-activated kinase 1, encoded by *Map3k7*) activation via Traf6 and Tab2/Tab3/Tak1 complexes^15^. Tak1 then activates the p38MAPK, JNK, and NF-κB pathways^16^, which further activate transcription factors NF-κB, c-Fos, c-Jun, MITF, and NFATc1 to turn on the expression of genes such as TRAP, Cstk, and Integrin β3 in osteoclasts. The importance of these molecules in osteoclasts was demonstrated by the findings that mice deficient for RANKL or RANK lack osteoclasts and show severe osteopetrosis^14^, and mice deficient for c-Fos and naturally occurring Mitf mutant mice (mi/mi mouse) also show osteopetrosis by halting osteoclast differentiation and/or fusion^17^,^18^.

Tak1 is a MAPKKK that can be activated by cytokines, growth factors, and T/B cell receptors^19^,^20^. TGFβ and BMPs activate Tak1 via Traf6, which further activates MAPK pathways, thus constituting the non-canonical signaling pathway of the TGFβ superfamily members^21^. In addition, Tak1 can be activated by DNA damage and was shown to have tumor suppressor activity^22^,^23^,^24^. Tak1 is an essential gene and conventional deletion of Tak1 leads to embryonic lethality^25^. Studies of conditional knockout mouse models indicate that Tak1 is an essential regulator of innate immunity, hematopoietic stem cell survival, energy sensing, and homeostasis of liver, smooth and cardiac muscles, and cartilage^26^,^27^,^28^,^29^,^30^,^31^.

Several groups reported that Tak1 is required for osteoclast survival and differentiation in vitro^32^,^33^. Tak1 has been ablated in mature osteoclasts with Cstk-Cre and the mice are severely osteopetrotic, very small and die soon after birth, making them not suitable for study of bone remodeling. In addition, Tak1 has been ablated in monocyte to study its function in macrophage and neutrophils, yet bone phenotypes were not reported^34^. Here we generated LysM-Cre; Tak1^f/f^ mice, which are viable, and studied bone remodeling in the adult mice. Our findings reveal a role for Tak1 not only in early to late stages of osteoclast differentiation via a combination of p38MAPK, Smad1, and NF-κB and related transcription factors, but also in skull/calvaria growth, articular cartilage growth, and in coupling to MSC cells. The drastic differences in body weight, bone parameters, and survival among LysM-Cre; Tak1^f/f^ mice, Cstk-Cre; Tak1^f/f^ mice, Rankl−/− mice, and Rank−/− mice strongly suggest that Cstk must be expressed in other cell types in addition to osteoclasts, and that the phenotypes of Cstk-Cre; Tak1^f/f^ mice may be caused by combination of defects in osteoclasts and non-osteoclast cells. Similar concerns were also raised by another recent study^35^. Thus, LysM-Cre; Tak1^f/f^ mice might present an animal model more suitable than Cstk-Cre; Tak1^f/f^ mice to study the function of Tak1 in bone remodeling.

## Methods

### Generation of conditional knockout of Tak1 in osteoclast precursors

The Tak1^f/f^ mice were generated in Michael D. Schneider's lab at National Heart and Lung Institute, Imperial College London United Kingdom^30^. The Lysozyme M-Cre (LysM-Cre) mice were purchase from The Jackson Laboratories. Genomic DNA was extracted from mice tails and was used for genotyping. Two-month-old male mice were used for bone histomorphometry experiments. All mice were housed in a pathogen-free facility. Animal experimentation in this study was carried out in accordance with recommendations in the National Research Council Guide for Care and Use of Laboratory Animals, with the protocols approved by the Institutional Animal Care and Use Committee of Shanghai, China [SYXK (SH) 2011–0112].

### Radiographic imaging

The mice were anesthetized by intraperitoneal injection of 0.375 mg/kg tribromoethanol dissolved in 2-methyl-2-butanol. The mice were then positioned properly and the radiographs of whole mouse were taken using Faxitron (MX-20 Specimen Radiography System) for qualitative evaluation. The X-ray images of the mutant and control mice of the same age and same gender were compared.

### Villanueva-goldner's trichrome staining of skull bone sections

Two-month-old mice were euthanized and mouse heads were scalded in hot tap water for 30 sec. The skin was carefully peeled off with forceps. Then the specimens were washed with PBS and fixed in 4% paraformaldehyde (PFA) for 24 hrs, which were put into decalcifying solution (15% EDTA) for 45 days until the bone was soft. The skull was then dehydrated twice with 70% ethanol for 20 min each, twice with 95% ethanol for 30 min each, and twice with 100% ethanol for 30 min each, followed by twice in xylene for 1 5 min each, in xylene/paraffin for 60 min at 58°C, three times in paraffin for 60 min each at 60°C, and then embedded with paraffin. The bone was sectioned at 5 μm, which was stained using Villanueva-goldner's one step trichrome method.

### Bone histomorphometry

Bone histomorphometry analysis was carried out following a protocol that has been previously described^36^,^37^. Briefly, seven days before the mice were sacrificed for histomorphometry analysis, calcein was subcutaneously injected at 0.2 mg/20 g body weight. One day before sacrificed, calcein was injected again. The femurs were removed and fixed in 4% PFA overnight and then stored in 70% alcohol for future experiments. The bones were dehydrated in 95% ethanol overnight, 100% ethanol for 5 hrs, 100% ethanol again overnight, which were then changed to acetone for 5 hrs, vacuumed for 30 min, transferred into xylene overnight, and vacuumed for 30 min. The bones were then embedded with resin, sliced and stained with Villanueva-goldner's one step trichrome method. All bone-specific parameters were measured and expressed in units following the guidelines established by the American Society for Bone and Mineral Research histomorphometry nomenclature committee using OsteoMeasure software (OsteoMetrics Inc).

### TRAP staining

Bone samples were decalcified for 4 weeks in 10% Ethylenediaminetetraacetic acid (EDTA) which were changed every 2 days, and then dehydrated in graded concentrations of alcohol from 70% to 100%. Following dehydration, samples were dealcoholised in xylene for 5 hours before being embedded in paraffin, which were cut into 5 μm sections. TRAP staining was carried out using Acid Phosphatase, Leukocyte TRAP Kit (387A, Sigma-Aldrich, St. Louis, USA) according to manufacturer's protocol. The slices were counter-stained with 0.05% Methyl Green and mounted with neutral gum.

### Measurement of bone resorption markers

Deoxypyridinoline in the urine was determined to evaluate the bone resorption rate, following the procedure recommended by MicroVue (Quidel), which was normalized to the urine levels of creatine. The concentration of calcium and inorganic phosphorus in the serum were determined using Calcium Liquicolor (Stanbio) and PHOS (FosunLong March Medical Science Co., Ltd) respectively.

### In vitro osteoclastogenesis and TRAP analysis

Mice bone marrow was flushed out with α MEM medium from the femurs and tibias. To induced the osteoclast differentiation, isolated bone marrow cells were first cultured in α MEM medium with 10% FBS at 37°C for 24 hrs. The floating cells were collected and seeded into 96-well plate at 1 × 10^6^/well, and were cultured in the presence of 20 ng/ml M-CSF and 40 ng/ml RANKL in α MEM medium with 10% FBS. The medium was changed every 3 days. After 7 days the cells were fixed and stained for TRAP using Acid Phosphatase, Leukocyte TRAP Kit (Sigma) following the manufacturer's protocol. Then we quantitated TRAP positive multinucleated osteoclast (≥3 nuclei).

### Immunohistochemistry staining

Detection of fibroblasts with anti-FSP-1 antibodies was performed on paraffin sections of calvarial bones by immunohistochemistry staining. Tissue sections were processed with an antigen retrieval step before they were incubated with anti-FSP-1 antibodies (Abcam) overnight at 4°C. These slides were then washed three times with PBS, incubated with horseradish peroxidase-labeled anti-rabbit antibody for 30 min at 37°C. The horseradish peroxidase was developed with diaminobenzidine tetrahydrochloride and hydrogen peroxide in PBS. Control sections were incubated without primary antibody.

### Mesenchymal stromal cell colony forming unit assay and ALP staining

Colony forming unit assay was carried out following a protocol that has been previously described^38^. Bone marrow was flushed out with αMEM medium (without serum) from the femurs and tibias and was collected in 50 ml falcon tube. Single cell culture was made through repeated passage through 19-gauge needles, which was then centrifuged for 3 min at 1000 rpm. The cells were resuspended in complete αMEM and plated at 5 × 10^6^ per well in 6-well plates at 37°C for 24 hrs. The floating cells were discarded. Fresh complete αMEM medium was changed every 3 days. Seven days later, the plates were washed with cold PBS, fixed with 4% PFA for 30 min, washed with ddH2O twice, and stained with an ALP detection kit (Sigma). The stained areas were measured using a densitometry system.

### Co-culture of MSC and monocyte/osteoclast

Co-culture experiments were carried out following a protocol that has been previously described^39^. Briefly, primary MSC were plated at 5 × 10^4^ per well in 24-well plates for 24 hrs. Upon confluency, freshly bone marrow monocytes were then plated on top of the MSC cells and cultured for 5 more days. The plates were then fixed and stained for ALP.

### Quantitative PCR

Cells were collected 3 days later after M-CSF and RANKL were added to induce osteoclast differentiation and total RNA was isolated from the cells with Trizol reagent (Invitrogen). Reverse transcription was performed using Transcriptor First strand cDNA synthesis kit (Roche) with random anchored-oligo(dT)18 primers. Real-time PCRs were performed using FS Universal SYBR Green Master Premix (Roche). Quantification was normalized to the amounts of endogenous GAPDH. The primers used for real-time PCR were: c-Fos, forward-CTGGTGCAGCCCACTCTGGTC, reverse- CTTTCAGCAGATTGGCAATCTC; NFATc1, forward- CTCGAAAGACAGCACTGGAGCAT, reverse-CGGCTGCCTTCCGTCTCATAG; TRAP, forward-GCTGGAAACCATGATCACCT, reverse-GAGTTGCCACACAGCATCAC; Mitf forward-CCCTCTCACCTGTTGGAGTCA, reverse-CCGTTTCTTCTGCGCTCATAC; Integrin β3, forward- TATGTGGTGGAAGAGCCTGAGTG, reverse-TCTTTATACAGCGGGTTGTTTGC; PU.1, forward-GATGGAGAAGCTGATGGCTTGG, reverse-TTCTTCACCTCGCCTGTCTTGC; GAPDH, forward-CCACAGTCCATGCCATCAC, reverse-CATACCAGGAAATGAGCTTGAC.

### Western blot analysis

Cells or spleen tissues were lysed with lysis buffer (200 mM Tris–HCl (pH 7.5), 1.5 M NaCl, 10 mM EDTA, 25 mM sodium pyrophosphate, 10 mM glycerolphosphate, 10 mM sodium orythovanadate, 50 mM NaF, 1 mM PMSF, 0.5% Triton, in combination with protein inhibitor cocktail). Twenty micrograms of protein lysates of each sample was subjected to SDS-PAGE analysis and transferred onto nitrocellulose membranes. Blots were incubated with the specific primary antibodies overnight at 4°C. After being washed three times for 15 min each with TBST (TBS + 0.1% Tween 20), blots were incubated with horseradish peroxidase-conjugated secondary antibody (Sigma) and visualized by chemiluminescence (Protein Simple).

### Data analysis

Statistical comparisons were performed using unpaired Student's two tailed t test, with p values < 0.05 considered statistically significant when compared to control mice. For bone histomorphometry analysis, 8 mutant and 8 control mice were used. For ex vivo studies, 3 pairs of mice were used. For experiments like western blot analysis, cells from several mice were pooled.

## Results

### Generation of monocyte-specific Tak1 knockout mouse

**Figure 1:**
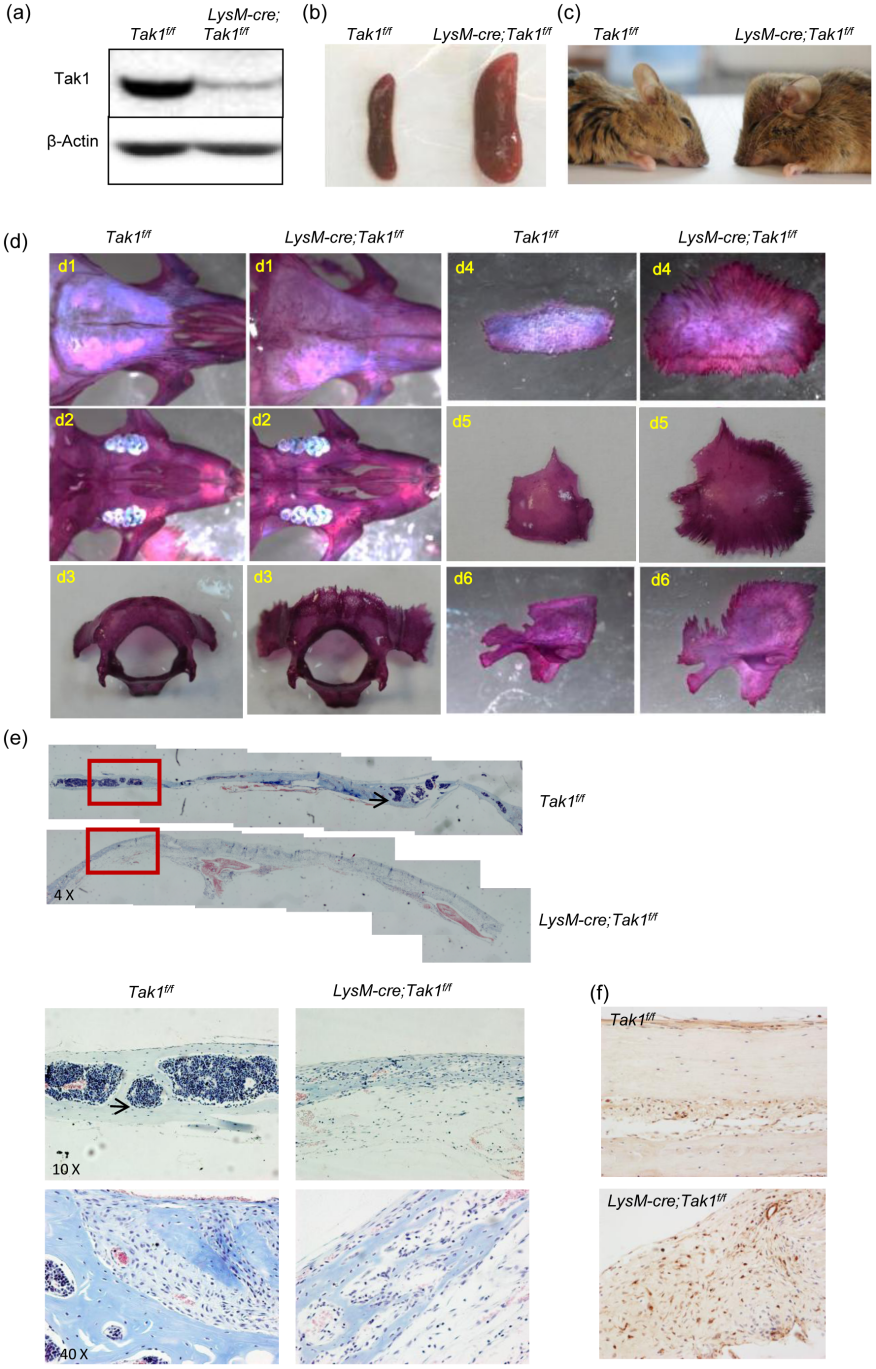
LysM-Cre; Tak1^f/f^ mice showed splenomegaly and skull overgrowth. (a) Western blot result showed that Tak1 was deleted in monocytes/osteoclasts of LysM-Cre; Tak1^f/f^ mice. Monocytes were isolated from LysM-Cre; Tak1^f/f^ and control mice and were induced to differentiate into osteoclasts by RANKL and M-CSF for 3 days. The cells were then harvested and cell lysates were analyzed with Western blot to determine the protein levels of Tak1. For the full-length blots see Supplementary Figure S1. (b) LysM-Cre; Tak1^f/f^ mice developed splenomegaly. Spleens were dissected out from 2 month-old LysM-Cre; Tak1^f/f^ and control mice. (c) The abnormal morphology of the skull of the adult LysM-Cre; Tak1^f/f^ mice. (d) LysM-Cre; Tak1^f/f^ mice show enlarged skull bones. Pictures were taken from 2 month-old LysM-Cre; Tak1^f/f^ and control mice. d1, the frontal bone; d2, palatoschisis; d3, occipital bone; d4, verge-interparietal bone; d5, verge-parietal bone; d6, temporal bone frontal bone. (e) Villanueva-goldner's trichrome staining of the calvarial bones of LysM-Cre; Tak1^f/f^ and control mice. Calvarial bones were decalcified, embedded in paraffin, sectioned, and stained with Villanueva-goldner's trichrome staining. Images of 4×, 10×, and 40× were shown in upper, middle, and bottom panels respectively. Arrows indicate bone marrow cavities. (f) LysM-Cre; Tak1^f/f^ mouse calvarial bones showed an increase in the number of FSP-1 positive fibroblasts. The decalcified calvarial bones were immunostained with anti-FSP-1 antibodies and detected with a DAB substrate kit.

Due to postnatal lethality of Cstk-Cre; Tak1^f/f^ mice^24^, we attempted to ablate Tak1 from osteoclast progenitor cells using the LysM-Cre mouse, which has been used to delete genes of interest in monocytes, macrophages, and granulocytes^34^,^40^, hoping to study Tak1 function in bone remodeling in adult mice. Western blot analysis of osteoclasts extracts confirmed that Tak1 was greatly reduced (Fig. 1a and Supplementary Fig. S1). The mutant mice also showed an enlarged spleen (Fig. 1b), as previously reported^34^. These results suggest that Tak1 is deleted in the mouse line. Unlike Cstk-mediated Tak1 knockout mice^32^, LysM-Cre; Tak1^f/f^ mice were born at expected Mendelian frequency and the pups and adult mice have similar body weights as control littermates (Supplementary Fig. S2). They are fertile and have limbs with similar length to those of control mice (Supplementary Fig. S2). Based on the fact that Rank−/− mice and Rankl−/− mice, which have almost no osteoclasts, appear only slightly smaller and do not show postnatal lethality^14^,^41^, our results imply that Cstk might be expressed in osteoclasts as well as non-osteoclast cells, and that the runtedness and postnatal lethality of Cstk-Cre; Tak1^f/f^ mice might be a result of defects of non-osteoclast cells. As such, LysM-Cre; Tak1^f/f^ mice might present an animal model more suitable to study the function of Tak1 in bone growth and remodeling.

### Skull and articular cartilage anomaly in monocyte-specific Tak1 knockout mouse

Some of the mice (5 out of 20 mice) showed visible anomaly of the skull, which was not obvious in newborn mice, but gradually showed up with age (Fig. 1c and Table 1). The mice with heterozygous deletion of Tak1 did not show this skull phenotype. Dissecting of the skull revealed that the frontal bone, palatoschisis, occipital bone verge, interparietal bone, verge-parietal bone, and temporal bone of adult knockout mice were much bigger than those of control adult mice (Fig. 1d). The calvarial bone in the mutant mice also looked bigger than that of the control mice (Fig. 1e). Section of calvarial bones revealed an increase in cellularity and a lack of bone marrow cavities in LysM-Cre; Tak1^f/f^ mice (Fig. 1e). Immunohistochemistry staining revealed that the mutant calvarial bones showed an increase in cells positive for fibroblast-specific protein-1 (FSP-1) (Fig. 1f), suggesting that deletion of Tak1 in monocytes/osteoclasts facilitates the proliferation of fibroblasts in calvarial bones.

**Figure 2:**
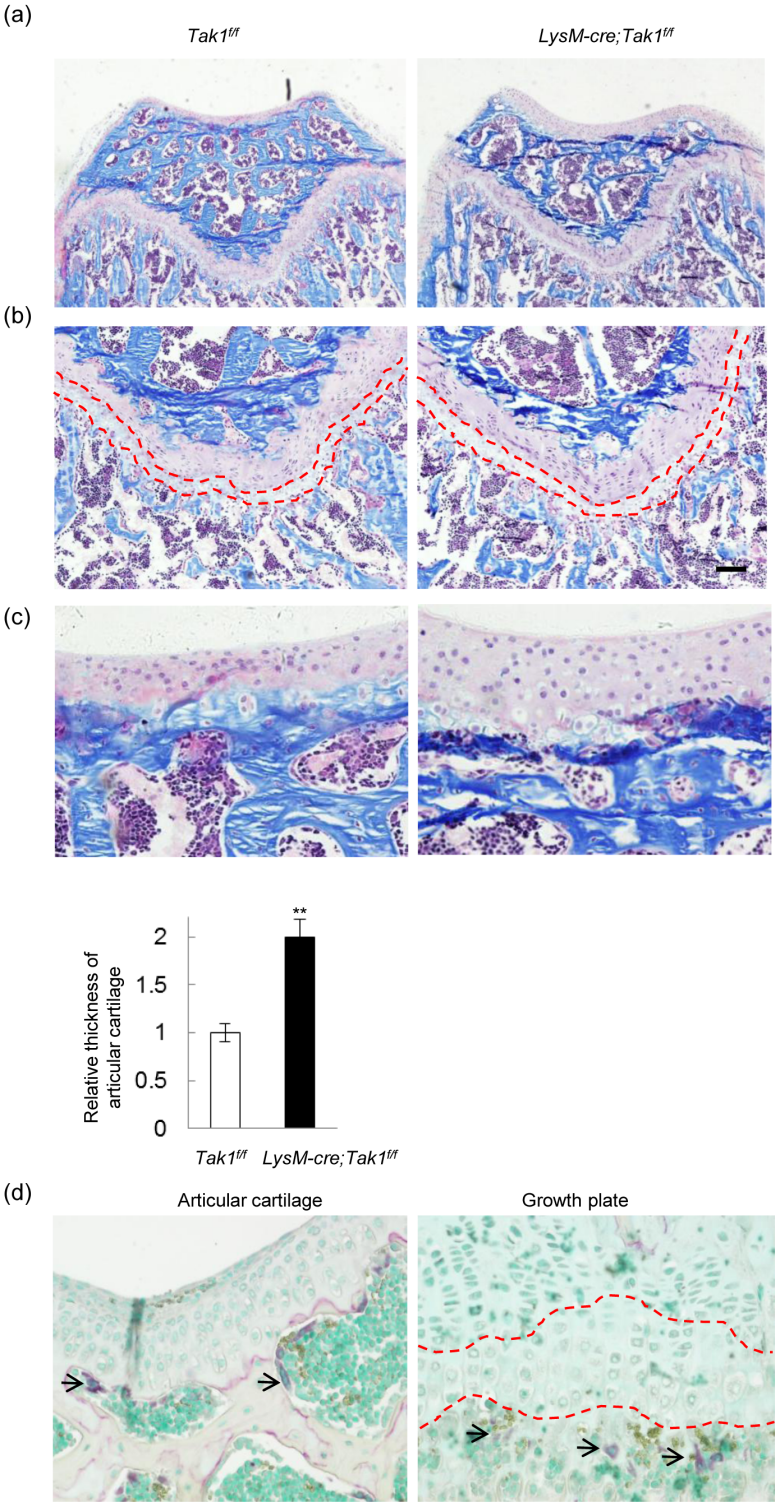
LysM-Cre; Tak1^f/f^ mice showed defects in articular cartilage. (a) The representative images of the distal end of femur bone that were taken from 2 month-old LysM-Cre; Tak1^f/f^ and control mice. The bones were decalcified, embedded in paraffin, sectioned, and stained with Villanueva-goldner's trichrome method. (b) LysM-Cre; Tak1^f/f^ mice showed normal growth plate in femur bones. Dashed lines indicate the hypertrophied and degenerated chondrocyte zones. (c) LysM-Cre; Tak1^f/f^ mice showed thickened articular cartilage. The bones were the same as described in Fig. 2b. Bottom panel: Quantitation data of the thickness of articular cartilage. N = 8. **p < 0.01 when the value of mutant mice was compared to that of control mice. (d) TRAP staining of femur bones shows that osteoclasts are present in the underchondral bone area of the articular cartilages (Left panel) and the trabecular bone region underneath the growth plates (Right panel). Arrows indicate TRAP positive osteoclasts. Dashed lines indicate the hypertrophied and degenerated chondrocyte zones.

In addition, section of the femurs revealed that the growth plates appeared normal in LysM-Cre; Tak1^f/f^ mice (Fig. 2a and 2b), yet the articular cartilage in all LysM-Cre; Tak1^f/f^ mice is thicker than that of normal mice (Fig. 2a and 2c), suggesting that Tak1 in monocyte/osteoclast might affect chondrocyte proliferation and/or differentiation at the articular surface. The lack of alteration in growth plate may be due to the fact that osteoclasts, which are present in metaphysis and diaphysis in long bones, are separated from the proliferation zone of the growth plate by the chondrocyte hypertrophy zone and the degeneration zone, whereas articular chondrocytes, which are not organized into zone-structures like epiphysis, are in close contact with osteoclasts in the bone end plate (Fig. 2d).

### Osteopetrosis phenotypes of adult monocyte-specific Tak1 knockout mouse

**Figure 3:**
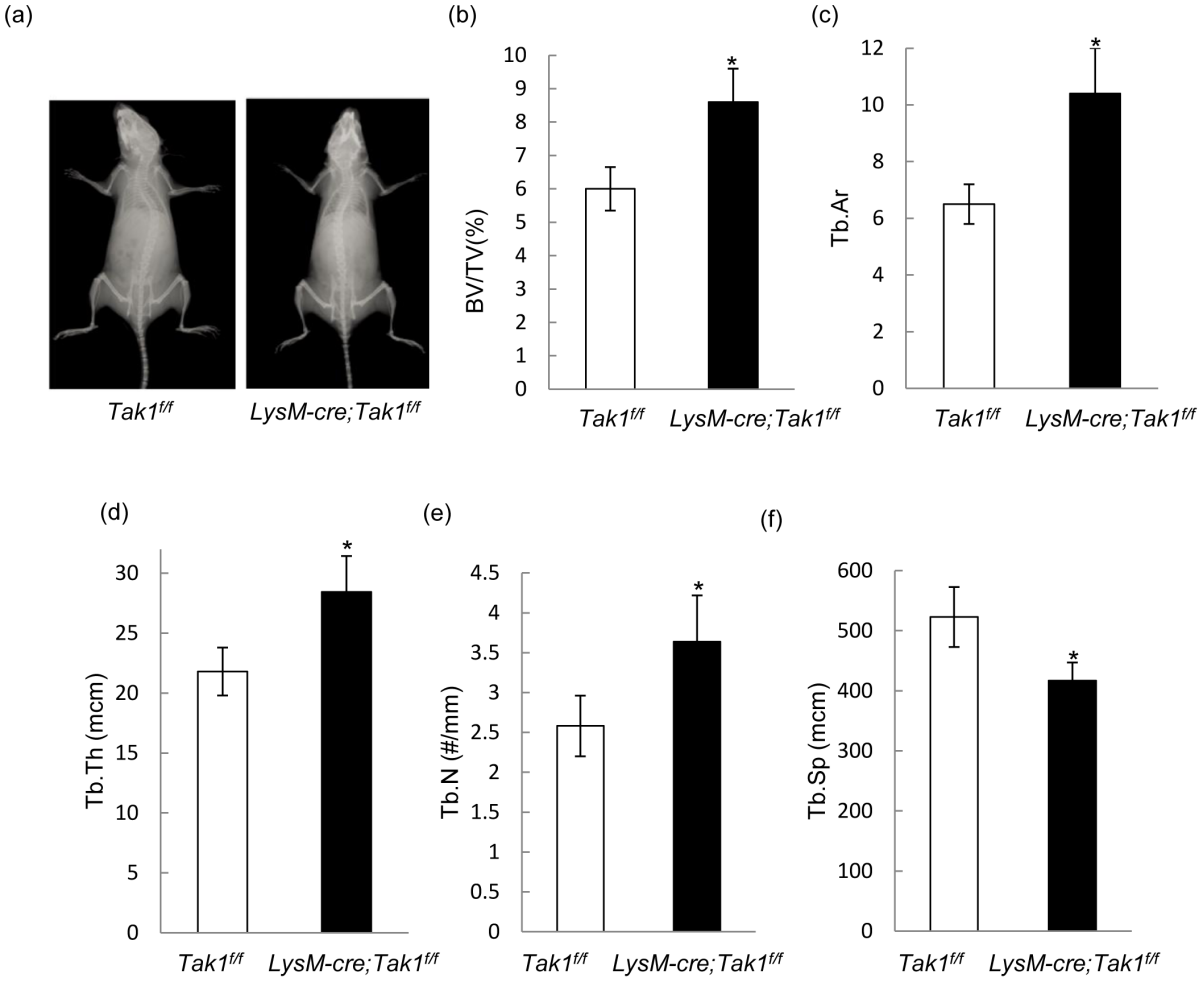
LysM-Cre; Tak1^f/f^ mice showed osteopetrosis phenotype. (a) Radiographic images of 2 month-old LysM-Cre; Tak1^f/f^ and control mice. (b–f). Trabecular bone parameters in 2 month-old LysM-Cre; Tak1^f/f^ and control mice. BV/TV (b). Tb area (c). Tb thickness (d). Tb number (e). Tb separation (f). The femurs were embedded with resin, sliced and stained with villauneva-goldner's one step trichrome method. All bone-specific parameters were measured and expressed in units following the guidelines established by the American Society for Bone and Mineral Research histomorphometry nomenclature committee using OsteoMeasure software (OsteoMetrics Inc). N = 8. *p < 0.05 when the value of mutant mice was compared to that of control mice.

To determine whether osteoclast-specific deletion of Tak1 affects bone remodeling in vivo, we radiographically examined 2 month-old LysM-Cre; Tak1^f/f^ and control littermates. It was obvious that the mutant mice showed a modest increase in overall bone mineral density (Fig. 3a), yet heterozygous deletion of Tak1did not show this phenotype (data not shown). We then performed bone histomorphometry analysis of 2-months old male LysM-Cre; Tak1^f/f^ and control mice. It was found that the bone mass was increased in the Tak1 deficient mice, with an increase in BV/TV, trabecular area, trabecular number, trabecular thickness, but a decrease in trabecular separation (Fig. 3b–3f). Collectively, these data suggest that LysM-Cre; Tak1^f/f^ mice showed an osteopetrotic phenotype. However, the increase in bone mass in LysM-Cre; Tak1^f/f^ mice compared to control littermates is much less than the increase in Cstk-Cre; Tak1^f/f^ mice (46% vs 400%), but is closer to the increase (about 100%) in bone mass/density reported in Rankl−/− mice^14^. Since monocyte-specific Tak1 deletion will be passed on to osteoclasts, the bone phenotypes of LysM-Cre; Tak1^f/f^ mice in theory should be similar to, if not worse than, Cstk-Cre; Tak1^f/f^ mice. The drastic increase in bone mass observed in neonatal Cstk-Cre; Tak1^f/f^ mice may be related to the extreme small bone size^32^.

### Bone formation and resorption of monocyte-specific Tak1 knockout mouse

**Figure 4:**
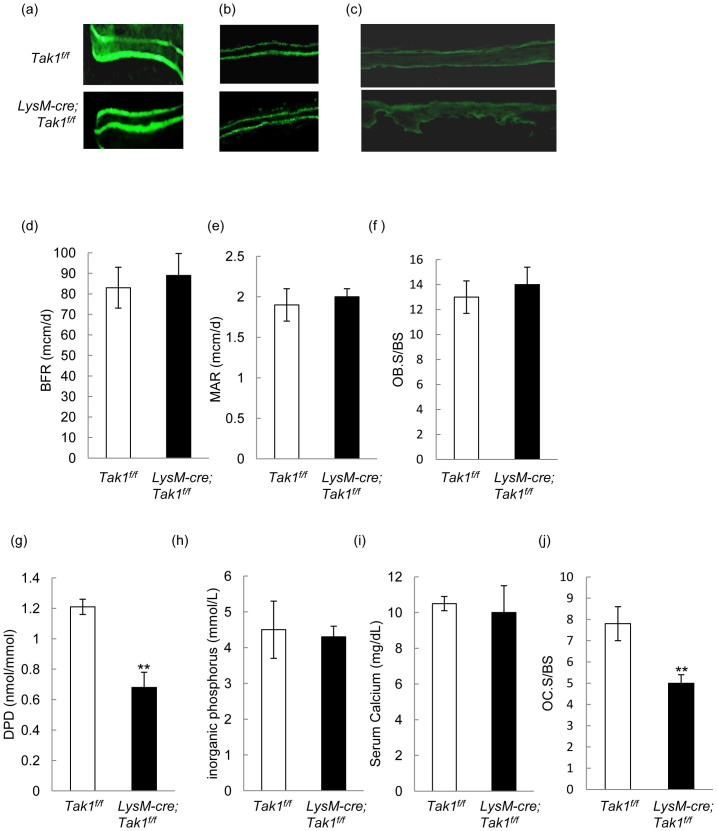
Bone formation and resorption parameters in LysM-Cre; Tak1^f/f^ mice. (a) Calcein double labeling in femur of 2 month-old LysM-Cre; Tak1^f/f^ and control mice. The mice were injected with Calcein twice with an interval of 7 days. The mice were sacrificed 1 day after the second injection. The femur bones were embedded, sectioned, and the images were taken using a Nikon microscope. (b) Calcein double labeling in the frontal bones of LysM-Cre; Tak1^f/f^ and control mice. (c) Calcein double labeling in the calvarial bones of 2 month-old LysM-Cre; Tak1^f/f^ and control mice. (d) Adult LysM-Cre; Tak1^f/f^ mice showed no change in bone formation rate (BFR). (e) Adult LysM-Cre; Tak1^f/f^ mice showed no change in mineral apposition rate (MAR). (f) Adult LysM-Cre; Tak1^f/f^ mice showed no change in the number of osteoblasts. (g) LysM-Cre; Tak1^f/f^ mice showed a decrease in urine DPD. The ELISA assays were performed on urine collected from mutant and control mice. N = 8. **p < 0.01 when the value of mutant mice was compared to that of control mice. (h) LysM-Cre; Tak1^f/f^ mice showed a normal level of serum Pi. (i) LysM-Cre; Tak1^f/f^ mice showed a normal level of serum Ca^2+^. (j) LysM-Cre; Tak1^f/f^ mice showed a decrease in the osteoclast surface per bone surface. N = 8. **p < 0.01 when the value of mutant mice was compared to that of control mice.

Increased bone mass could be caused by increased bone formation, decreased bone resorption, or both, which are usually studied in 2 month-old mice. We then carried out calcein double label experiments and found that the distance between the two labels, which is a reflection of bone formation rate, was not altered in the femurs of LysM-Cre; Tak1^f/f^ mice (Fig. 4a), nor was the calcein labels altered in the frontal bones or the calvarial bones (Fig. 4b and 4c), which were enlarged in LysM-Cre; Tak1^f/f^ mice (Fig. 1d and 1e). Histomorphometric analysis confirmed that Tak1 ablation in monocytes/osteoclasts did not alter the bone formation rates or mineral apposition rates in the femurs (Fig. 4d and 4e), nor did it affect bone formation rates (147.56 ± 15.88 for WT vs. 142.56 ± 22.37 for the mutant) or mineral apposition rates (1.131 ± 0.112 for WT vs. 0.987 ± 0.193 for the mutant) in the frontal bones. Moreover, the mutant mice did not show any significant change in the number of osteoblasts per bone surface in the femurs (Fig. 4f). These results suggest that deletion of Tak1 in monocytes does not significantly alter bone formation in 2 month-old mice. Thus, the increase in the size of skull bones is not accompanied by thickening of the mineralized bones. How Tak1 ablation in monocytes/osteoclasts affects the size of skull bones warrants further investigation.

We then analyzed bone resorption markers and found that urine deoxypyridinoline (DPD), a product of bone resorption, was decreased in LysM-Cre; Tak1^f/f^ mice (Fig. 4g), yet the concentration of the inorganic phosphorus and calcium did not change much (Fig. 4h and 4i). Note Rankl−/− mice did not show much change in serum levels of calcium and phosphorus either, although they showed osteopetrosis^14^. Moreover, the number of osteoclasts was significantly reduced in LysM-Cre; Tak1^f/f^ mice (Fig. 4j). It is worth to mention that the degrees of decrease in DPD levels and decrease in the number of osteoclasts fall in the range of the increase in bone volume in LysM-Cre; Tak1^f/f^ mice, suggesting that osteopetrosis of LysM-Cre; Tak1^f/f^ mice is caused by decreased bone resorption due to decreased osteoclast numbers.

### Tak1 regulates osteoclast differentiation at multiple stages via multiple signaling pathways

**Figure 5:**
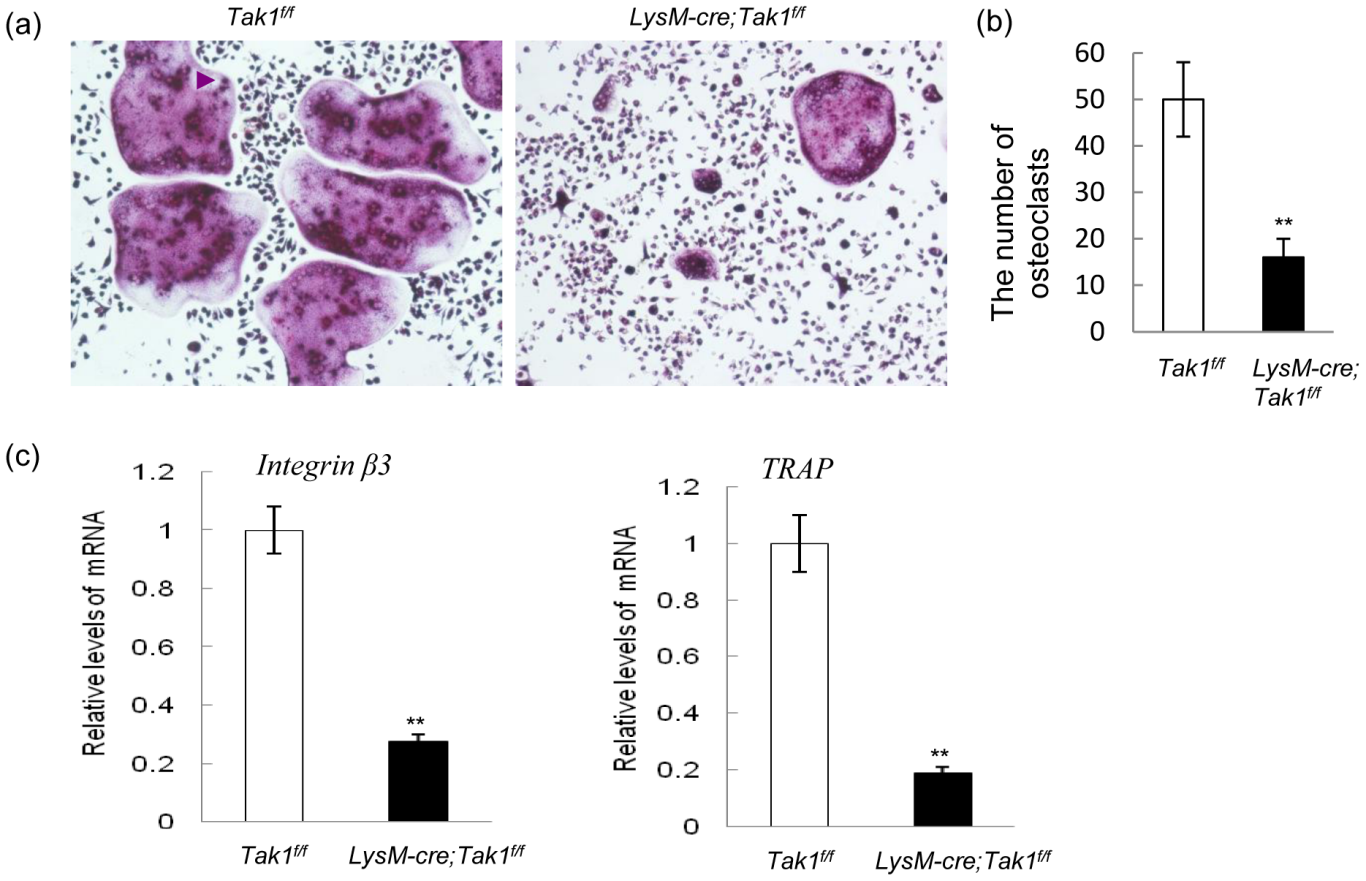
LysM-Cre; Tak1^f/f^ mice showed a defect in osteoclastogenesis. (a) LysM-Cre; Tak1^f/f^ monocytes showed a reduction in differentiating into TRAP positive osteoclasts. Bone marrow suspension cells were cultured in the presence of RANKL and M-CSF for 7 days and were then stained for TRAP. (b) Quantitation data of TRAP positive multinucleated osteoclast (≥3). N = 3. **p < 0.01 when the value of mutant cells was compared to that of control cells. (c) LysM-Cre; Tak1^f/f^ monocytes showed reduced expression of Integrin β3 and TRAP. Osteoclast cultures were collected three days after addition of RANKL and M-CSF and total RNA was isolated from these cultures and realtime PCR was carried out to determine the mRNA levels of Integrin β3 (left panel) and TRAP (right panel). N = 3. **p < 0.01 when the value of mutant cells was compared to that of control cells.

Previous studies suggested that Tak1 positively regulates osteoclast maturation^32^. To confirm these findings, we isolated monocytes from adult LysM-Cre; Tak1^f/f^ and control mice, which were induced to differentiate into osteoclasts with M-CSF and RANKL. Osteoclasts were then stained for TRAP, a specific marker for osteoclasts. While mature multi-nuclear osteoclasts (≥3 nuclei) were successfully induced in the control group, only a few of Tak1−/− osteoclasts were generated in the same settings (Fig. 5a and 5b), yet the total number of TRAP positive cells (≥1 nucleus) was similar between WT and Tak1−/− cultures (data not shown), suggesting that the main difference is osteoclast differentiation. Realtime PCR analysis showed that osteoclast maturation marker TRAP and integrin β3 were expressed at much reduced levels in the absence of Tak1 (Fig. 5c). These results, together with the decrease in the number of osteoclasts in vivo, suggest that Tak1 plays a critical role in osteoclastogenesis.

**Figure 6:**
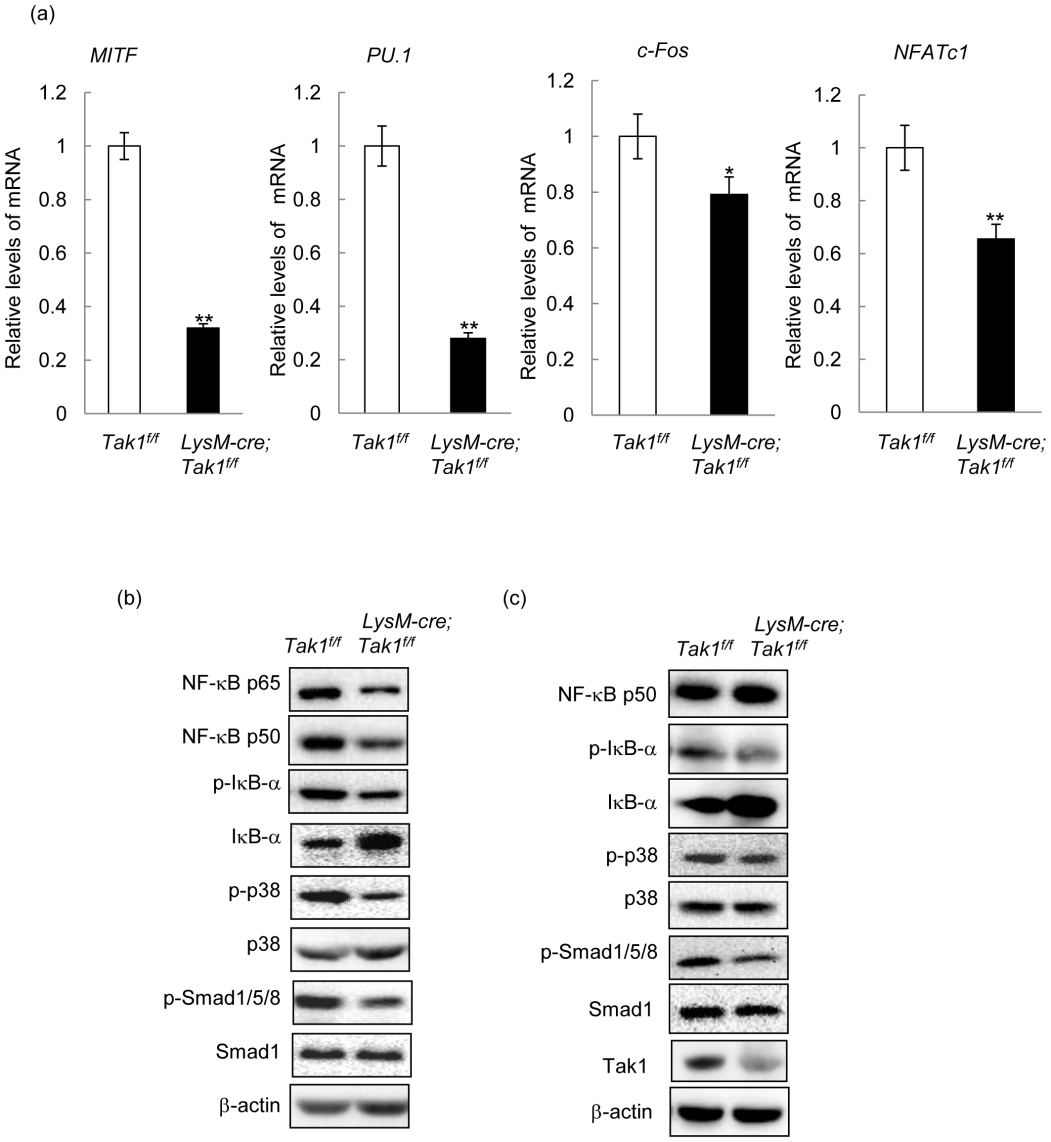
Tak1 regulates osteoclast differentiation via multiple transcription factors and signaling pathways. (a) The reduced mRNA levels of various transcription factors involved in osteoclast differentiation. Osteoclast cultures were collected three days after addition of RANKL and M-CSF and total RNA was isolated from these cultures and realtime PCR was carried out to determine the mRNA levels of these transcription factors. N = 3. *p < 0.05, **p < 0.01 when the value of mutant cells was compared to that of control cells. (b) Reduced activation of Tak1 downstream signaling pathways in Tak1 deficient monocyte/osteoclast cultures. Osteoclast cultures were collected three days after addition of RANKL and M-CSF and cells were harvested and Western blot was carried out to determine the levels of these activated signaling molecules. For the full-length blots see Supplementary Figure S3. (c) Analysis of the signaling events in the spleen of LysM-Cre; Tak1^f/f^ mice. Spleens were homogenized and the samples were analyzed by western blot to determine the activation the signaling molecules. For the full-length blots see Supplementary Figure S4.

Osteoclast differentiation was controlled by several transcription factors such as c-Fos and NF-κB. While freshly isolated monocytes express undetectable levels of these transcription factors, the addition of RANKL and M-CSF greatly induced their expression, yet, Tak1 deficient monocyte/osteoclast cultures showed a decrease in PU.1, MITF, NFATc1, and c-Fos, with c-Fos expression being less drastically reduced (Fig. 6a). Since PU.1 and MITF also act at the committed myeloid precursor^6^,^11^, the results suggest that Tak1 may play a role in early osteoclast differentiation stage before RANKL exerts it action.

We then analyzed the signaling pathways that are involved in osteoclast differentiation. We compared their activation in monocyte/osteoclast cultures after induction of the differentiation program. It was found that NF-κB molecules including p65 and p50 were down-regulated in the absence of Tak1 (Fig. 6b and Supplementary Fig. S3), so were p38MAPK activation and Smad1/5/8 activation, although to a much lesser extent (Fig. 6b and Supplementary Fig. S3), suggesting that Tak1 is required for full activation of these three pathways in monocytes/osteoclasts, as shown in other cells^42^,^43^. Again, Tak1 deficiency decreased but not abolished the activation of these signaling pathways, which is consistent with the degree of decrease in osteoclast differentiation. Given the well-established roles for p38MAPK-c-Fos and NF-κB in osteoclast differentiation, these results indicate that Tak1 might use both p38MAPK and NF-κB signaling pathways to regulate osteoclast differentiation. Recent studies also revealed an important role for BMP-Smad1 signaling in osteoclastogenesis^40^,^44^. The decreased Smad1/5/8 activation may also contribute to altered osteoclast differentiation of Tak1−/− monocytes. In addition, we also determined activation of above mentioned signaling molecules in the homogenates of spleen, which is much enlarged in the mutant mice (Fig. 1b), and found that Tak1 deficiency led to a slight decrease in the activation of p38MAPK and Smad1/5/8, but not NF-κB (Fig. 6c and Supplementary Fig. S4). While previous studies have suggested that Tak1 might have cell type-specific effects^34^, it is possible that the less obvious difference observed in the spleen is due to the fact that Lysozyme positive monocytes constitute only a small percentage of spleen mass and the Tak1 western blot results is masked by other cells in the spleen.

### Monocyte-specific deletion of Tak1 led to an increase in bone marrow mesenchymal stromal cells

**Figure 7:**
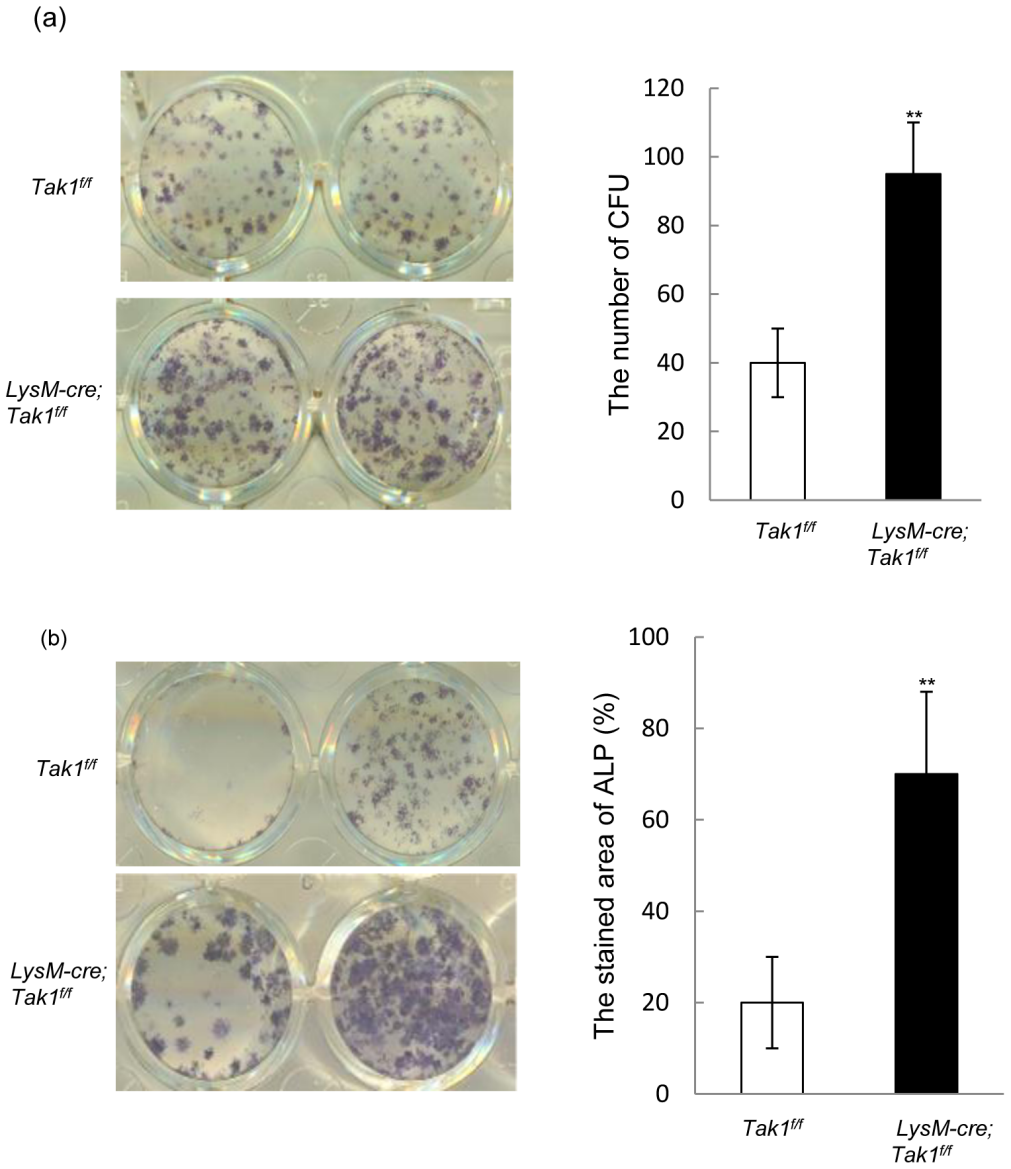
Tak1 deficient monocytes/osteoclasts showed increased activity to promote osteoblastogenesis. (a) LysM-Cre; Tak1^f/f^ mice showed an increase in the number of ALP positive CFU. Bone marrow cells were isolated and red blood cells were lysed. The cells were washed with PBS and counted. 5 × 10^6^ cells were plated into each well of the 6 well plates and were cultured in α-MEM with 10% serum. The plates were stained for ALP after plated for 7 days. Right panel: Quantitation data. N = 5. **p < 0.01 when the value of mutant cells was compared to that of control cells. (b) LysM-Cre; Tak1^f/f^ monocytes showed a better activity to support MSC osteogenic differentiation in co-culture assays. Wild type MSC cells were plated and cultured for 24 hrs. Freshly bone marrow monocytes isolated from LysM-Cre; Tak1^f/f^ and control mice were plated on top of the MSC cultures, without RANKL or M-CSF. After 5 days, the cells in suspension were washed off and the adherent MSC cells were stained for ALP. N = 3. **p < 0.01 when the value of mutant cells was compared to that of control cells.

Although LysM-Cre; Tak1^f/f^ mice did not show a significant change in bone formation, we found that the mutant mice showed an increase in mesenchymal stromal cells, which are the progenitor cells for osteoblast, chondrocyte, and adipocyte^4^. We isolated bone marrow cells from LysM-Cre; Tak1^f/f^ and control littermates, lysed red blood cells, and plated the same number of cells. After 7 days, the plates were stained for ALP, a marker for osteoblasts. It was found that LysM-Cre; Tak1^f/f^ mice showed an increase in the number and the size of colony forming units, a reflection of the number and proliferation of osteoprogenitor cells (Fig. 7a). The correlation between defective osteoclast differentiation and increased ALP positive CFU suggest that Tak1 in osteoclasts might inhibit osteoblastogenesis. To test this, we carried out a co-culture experiment of monocytes and MSC. The same number of wild type MSC were plated and cultured overnight, and the same number of bone marrow cells isolated from WT and LysM-Cre; Tak1^f/f^ mice were then added to the culture. These cells were cultured for 5 more days and then stained for ALP. It was found that Tak1−/− monocytes showed an increased ability to stimulate MSC osteogenic differentiation (Fig. 7b), likely under the stimulatory effects of BMP and Wnt molecules present in the fetal bovine serum. Collectively, these results revealed a role for osteoclast-expressed Tak1 in regulating osteoblastogenesis. Note that the increase in the number of MSC was not reflected in the number of osteoblasts and bone formation rate in vivo.

## Discussion

Osteoporosis, as well as osteosclerosis and osteopetrosis, arises when the balance between bone formation and bone resorption is disrupted^2^,^3^. Osteoclast-mediated bone resorption is a major target for the development of drugs for osteoporosis prevention and therapy^9^. So it is crucial to understand the signaling network that controls osteoclastogenesis. Our study of the monocyte-specific Tak1 knockout mice confirmed an important role for Tak1 in osteoclastogenesis and bone resorption in vivo. Yet the defects caused by Tak1 deletion is much less than mature osteoclast-specific deletion of Tak1 mediated by Cstk-Cre, which are unexpectedly more severe than those of Rankl−/− or Rank−/− mice^41^. Furthermore, while Cstk-Cre; Tak1^f/f^ mice show severe runtedness at birth and postnatal lethality, the LysM-Cre; Tak1^f/f^ mice appear normal in body weight and limb size, fertility, and look normal from p1 to 12 month of age except the skull phenotype. Given that Rankl−/− and Rank−/− mouse lines appear normal at birth and fertile and only develop runtedness after weaning, it is possible that runtedness and postnatal lethality of Cstk-Cre; Tak1^f/f^ mice are not a direct result of osteoclast defects. Instead, they are more likely to be caused by defects in other cells/tissues that also express Cathepsin K, as previously reported^35^.

Our study also revealed that monocyte-specific deletion of Tak1 could lead to an increase in the sizes of the skull bones, although with incomplete penetrance. The increase in the skull bone sizes is not accompanied by an increase in the thickness of bones or bone formation rates. Instead, an increase in the number of fibroblasts was observed in the calvarial bones. This phenotype is getting more obvious with age, suggesting that it is not a developmental defect, rather, it is a problem arisen during growth. It has also been reported that Tak1 is also involved in TGFBRII mutation-induced skull development defects^45^. These findings, taken together, suggest that Tak1 might participate in the signaling events that control skull development and growth. Yet, how Tak1 acts in osteoclasts to regulate skull bone growth needs further investigation.

Analysis of LysM-Cre; Tak1^f/f^ adult mice also revealed a role for Tak1 in connecting osteoclastogenesis and osteoblastogenesis. LysM-Cre; Tak1^f/f^ adult mice showed an increase in ALP positive colony forming units of bone marrow mesenchymal stromal cells, suggesting that the defect in osteoclast differentiation might influence the differentiation potential of MSCs. Indeed, we found that monocyte deficient for Tak1 showed an enhanced activity in promoting MSC osteogenic differentiation in co-culture assays, likely through growth factors/cytokines secreted by monocytes/osteoclasts. Surprisingly, we failed to observe an alteration in the number of mature osteoblasts or bone formation rate in the 2 month-old LysM-Cre; Tak1^f/f^ mice. This discrepancy can be explained by that the increased MSC cells may serve as reserves and are not in an active state, or that the effect of increased number of MSC cells may take time to show up. In addition, we found that monocyte-specific deletion of Tak1 led to an increase in the thickness of articular cartilage, suggesting that osteoclasts might also communicate with MSC cells at the articular surface to regulate chondrocyte differentiation. The fact that monocyte-specific Tak1 deletion did not affect chondrocytes at the growth plate could be explained by that the hypertrophied chondrocyte zone and the degenerated chondrocyte zone separate the proliferating chondrocytes from osteoclasts that are present in the metaphysis. Thus, Tak1 presence in monocytes/osteoclasts appears to affect differentiation of the neighboring MSCs into osteoblast or chondrocyte, suggesting that Tak1 is involved in communication between osteoclast precursors and MSC, likely via synthesizing and secreting cytokines and/or growth factors. Future efforts will be needed to identify these cytokines and growth factors.

Tak1, as a downstream signaling molecule of TGFβ and BMPs, has been shown to regulate chondrocyte proliferation and differentiation in cell-autonomous manners, likely via downstream MAPK and Smad1/5/8 pathways^42^,^43^,^46^. In addition, mature osteoblast-specific ablation of Tak1 also led to the development of cleidocranial dysplasia, with a reduction in the parietal and frontal bones mineralization^47^. This study and others' show that Tak1 is also a positive regulator of osteoclast differentiation and survival^32^. How does Tak1 regulate osteoclast differentiation? The observation that PU.1 and MITF were markedly down-regulated in Tak1−/− monocytes/osteoclasts suggests that Tak1 might play a role at early stage of monocyte differentiation, before RANKL puts forth its action^5^,^11^. Indeed, it has been reported that Tak1 plays an important role in the differentiation of macrophages and neutrophils, two other progenies of monocytes^34^. Furthermore, in response to RANKL, it seems that Tak1 deficiency led to a decrease in NF-κB, p38MAPK, and Smad1/5/8 activation. While NF-κB and p38 are well-established promoter of osteoclast differentiation, a role for BMP-Smad1 pathway in osteoclast differentiation needs to be further examined. Two recent studies implicated that BMP-Smad1 signaling regulates osteoclast differentiation. Deletion of BMPRIA with Cstk-Cre (at day 4 after induction of differentiation) enhanced osteoclast differentiation^44^, whereas LysM-Cre mediated ablation of BMPRII led to compromised differentiation^40^. We recently found that LysM-Cre mediated ablation of BMPRIA resulted in enhanced osteoclast differentiation (Li A. and Li B., unpublished results). Therefore it is possible that decreased Smad1/5/8 activation acts as a balancing mechanism to regulate osteoclast differentiation in LysM-Cre; Tak1^f/f^ mice. It is also possible that BMP-Smad1 signaling plays different roles at various stages of osteoclast differentiation. Mechanistically, BMP-Smad1 signaling might regulate osteoclast differentiation by interacting with the NF-κB pathway. It has been previously reported that Smad7, a target gene of BMP-Smad1 signaling, interacts with Tab2/3 and thus inhibits the Tak1-NF-κB activation^48^. On the other hand, Tak1 was reported to interact with various Smad molecules and this inhibits BMP-Smad1 signaling^41^,^49^,^50^.

In summary, this study revealed an important role for Tak1 in monocyte/osteoclast differentiation, which might be mediated by combined alterations of downstream signaling pathways, including Smad1, NF-κB, and p38MAPK, and is quantitatively different from the role revealed in mature osteoclasts. Moreover, Tak1 also plays a role in communication from monocytes/osteoclasts to MSC and regulates MSC differentiation into osteoblasts or chondrocytes, likely via paracrine secretion of signaling molecules. By doing that, Tak1 regulates the growth of skull bones and articular cartilage.

## Author Contributions

B.L. conceived the project and wrote manuscript. H.L. guided the research and put the data in order. B.Q. and Q.C. performed the experiments and analyzed the data (Figures 1–7). P.L., G.M. and X.G. assisted with the animal treatments and qPCR experiments (Figure 5B and Figure 6A). J.Y. guided the Bone histomorphometry experiment (Figures 3–4). M.X. and M.D.S. provided the Tak1^f/f^ mice. All authors reviewed the manuscript.

## Supplementary Material

Supplementary Information SUPPLEMENTARY

## Figures and Tables

**Table 1:** The fraction of LysM-Cre; Tak1^f/f^ mice that show abnormal skull phenotypes

Genotype	Mice with skull defect		Number of mice		Prevalence	Age
	Male	Female	Male	Female		
*Tak1^f/f^*	0	0	10	10	0 out of 20	2 month
*LysM-cre; Tak1^f/f^*	3	2	10	10	5 out of 20	2 month
