# Supplementary material for: Ablation of Tak1 in osteoclast progenitor leads to defects in skeletal growth and bone remodeling in mice
Source: Sci Rep. 2014 Nov 24;4:7158. doi: 10.1038/srep07158 (PMC4241509; doi:10.1038/srep07158)

Supplementary information

Ablation of Tak1 in osteoclast progenitor leads to defects in skeletal growth and bone remodeling in mice

Bing Qi[1 *], Qian Cong [2 *], Ping Li[2], Gang Ma[2], Xizhi Guo[2], James Yeh[2], Min Xie[3], Michael D. Schneider[4], Huijuan Liu[2, #], and Baojie Li[2, #]

Supplementary Figures

Figure S1 The full length blots for Fig. 1A

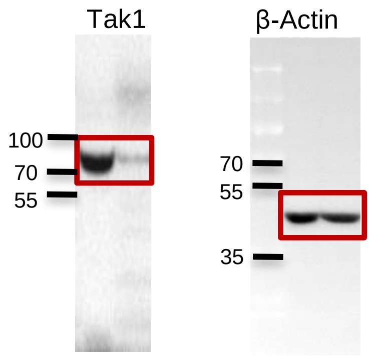

Figure S2. Adult LysM-Cre; Tak1f/f mice showed body weight (left panel) and limb length (right panel) comparable to control mice. N=8.

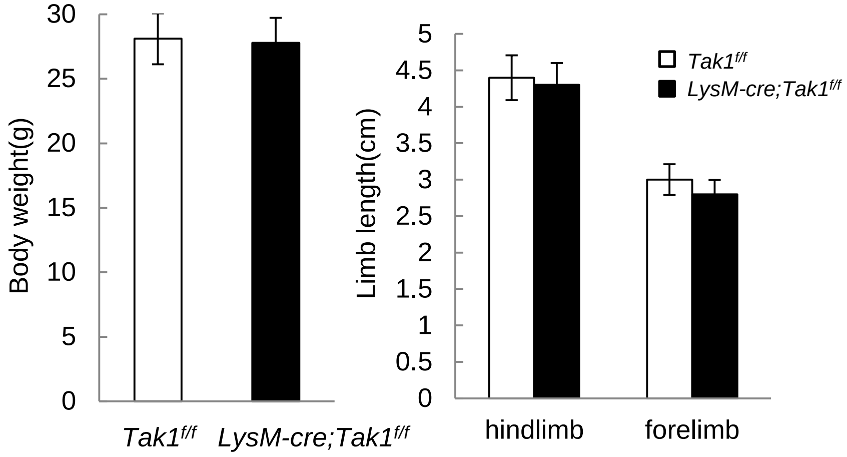

Supplementary Figures

Figure S3 The full length blots for Fig. 6B

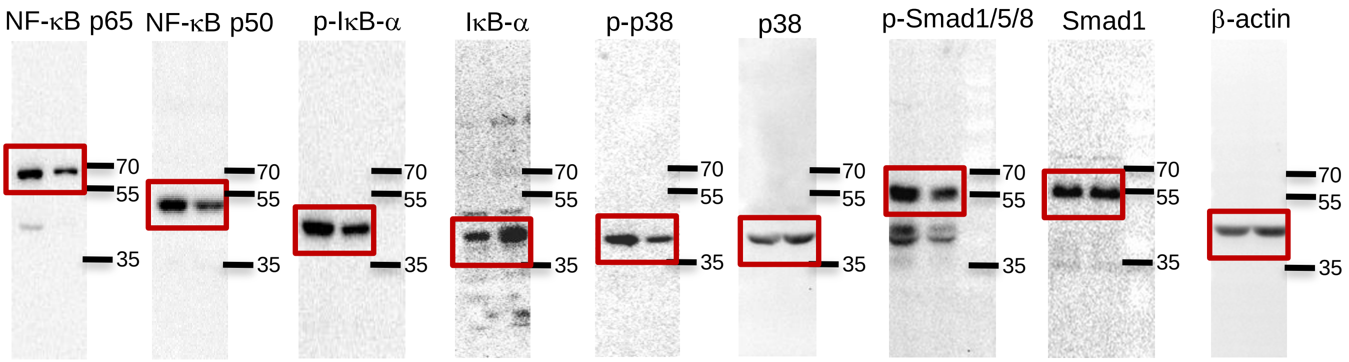

Supplementary Figures

Figure S4 The full length blots for Fig. 6C

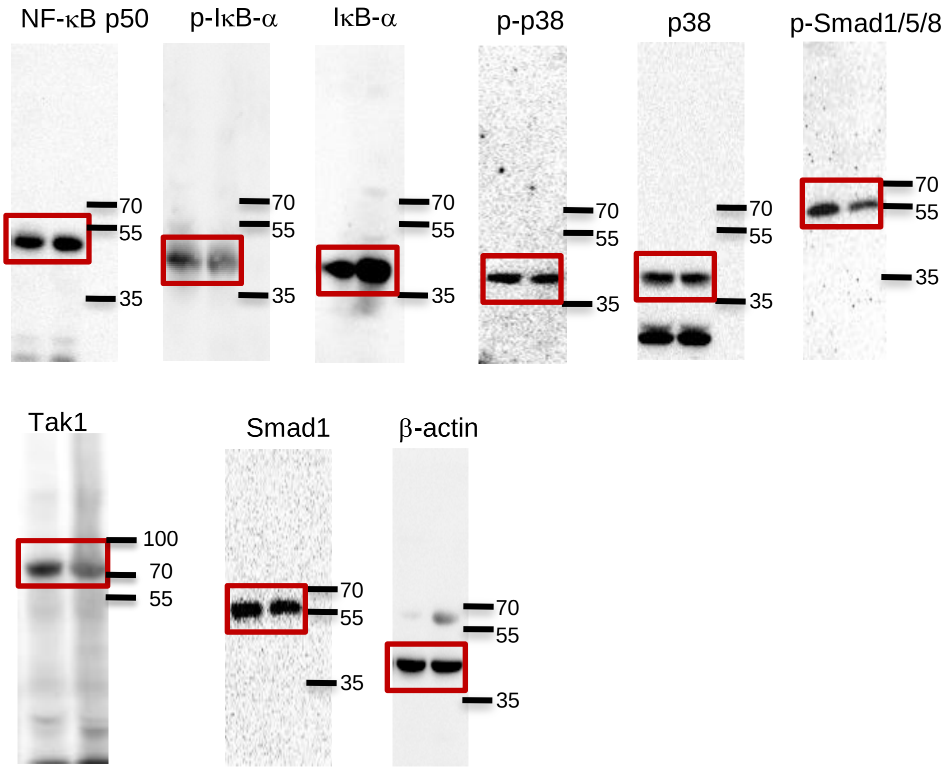

Supplement: Supplementary Information — SUPPLEMENTARY [file srep07158-s1.pdf]
